# Reciprocal relationships between paternal psychological distress and child internalising and externalising difficulties from 3 to 14 years: a cross-lagged analysis

**DOI:** 10.1007/s00787-020-01642-0

**Published:** 2020-09-17

**Authors:** Maria Sifaki, Emily Midouhas, Efstathios Papachristou, Eirini Flouri

**Affiliations:** Department of Psychology and Human Development, UCL Institute of Education, University College London, 25 Woburn Square, London, WC1H 0AA UK

**Keywords:** Child behaviour, Cross-lagged panel models, Millennium cohort study, Paternal psychological distress

## Abstract

Research shows that paternal psychological distress is associated with child emotional and behavioural difficulties. However, little is known about the direction of this association including whether it is bidirectional. The aim of this study was to explore the reciprocal relationships between paternal psychological distress and child emotional and behavioural problems longitudinally (at ages 3, 5, 7, 11 and 14 years) in a sample of 13,105 children (49% girls) who participated in the UK’s Millennium Cohort Study (MCS), a large-scale, nationally representative, longitudinal survey. Four domains of child problems (emotional symptoms, peer relations, conduct problems and hyperactivity/inattention) were measured with the Strengths and Difficulties Questionnaire and paternal psychological distress was measured with the Kessler K-6 scale. Data were analysed using cross-lagged path models. At all ages, paternal psychological distress predicted both subsequent child emotional symptoms as well as peer problems. Paternal psychological distress at child’s age 3 was related to more hyperactivity at age 5 and, at age 5, paternal psychological distress was associated with more conduct problems at age 7. At age 11, paternal distress was also related to age 14 conduct problems and hyperactivity. Child effects were fewer and were found mainly for behavioural problems. Notably, we found bidirectional links between paternal psychological distress and child peer difficulties, from 11 to 14 years. Paternal psychological distress appears to influence child behaviour more consistently than the converse. However, in early adolescence, there appears to be a reciprocal relationship between fathers’ mental health problems and children’s peer problems.

**Electronic supplementary material:**

The online version of this article (10.1007/s00787-020-01642-0) contains supplementary material, which is available to authorized users.

## Introduction

An extensive amount of research has demonstrated the damaging and long-term consequences of maternal psychological distress on child emotional and behavioural difficulties [1]. Other research suggests effects in the opposite direction such that child emotional–behavioural problems increase maternal psychological distress, or that maternal psychological distress and child problems may influence each other [2–5]. The literature has placed much less emphasis on the relationship between fathers’ psychological distress and children’s emotional and behavioural problems [6]. It is important, however, to explore further the influence of paternal well-being on children, as fathers nowadays are more involved in child-rearing than in the past [7]. Hence, paternal psychological distress might be a greater risk factor for child outcomes than in the past and knowing how and when psychological distress may exert its effect could help prevent its impact. Indeed, emerging evidence indicates that fathers’ psychological distress adversely affects children’s emotional and behavioural problems [8–10]. Two meta-analytic studies have reported small yet significant effect sizes [8, 9]. A small amount of research has begun to investigate the converse—the role of child behaviour in paternal psychological distress—and indeed, whether paternal and child difficulties simultaneously influence each other [11–13].

Although the research into reciprocal associations between child difficulties and paternal psychological distress is limited, there has been much related research suggesting that such associations may be plausible. For example, child difficulties provoke marital tension in both parents, which in turn adversely impacts their well-being [14]. There is more research, however, on the opposite direction of the relationship, with many studies showing that father’s psychological distress is related to child emotional and behavioural problems, even after controlling for many important confounding variables, including maternal psychological distress and socio-economic disadvantage [15–25]. Most of these studies have utilised community samples and longitudinal approaches to evaluate the long-term consequences of paternal distress. Typically, however, such research examines father effects only and usually from infancy to early childhood [15, 17, 19, 21, 22, 24]. Longitudinal research looking into adolescence [16, 20, 23] or on the reciprocal influences of paternal and child mental health is limited.

Reciprocal associations between father’s psychological distress and child’s behaviour have been explored in three studies, to our knowledge [11–13]. In particular, Gross et al. [11] examined the transactional links between child difficulties and paternal (and maternal) depressive symptoms at child’s ages 2 and 4 using data on 731 parent–child dyads participating in a US-based longitudinal intervention study. They found that paternal symptoms at age 2 predicted child internalising difficulties at age 4 but child noncompliance at age 2 did not predict paternal depressive symptoms at age 4. The child measures at ages 2 and 4 capture different behavioural constructs and, therefore, limit the rigour of the prior behaviour adjustment (at age 2) when examining paternal effects on behaviour at age 4. The authors also explored maternal depressive symptoms and child difficulties in an equivalent model, finding that maternal depressive symptoms influenced both internalising and externalising problems. However, paternal depressive symptoms influenced only internalising problems.

The second study, using data from the National Institute of Child Health and Human Development (NICHD) Study of Early Child Care [12], explored the reciprocal links between father’s distress and offspring internalising and externalising difficulties, at multiple time-points, from the age of 4.5 years to the age of 15 years. It also ran an identical model of mother’s distress and both types of child difficulties. Paternal distress predicted externalising difficulties in early childhood, but there were both father effects and child effects in adolescence. Regarding the internalising domain, in line with Gross et al. [11]’s findings, fathers’ distress predicted offspring difficulties consistently. Additionally, transactional associations were found during early adolescence, from 11 to 12 years [12]. Later in adolescence, this was the case for females only, as males’ difficulties were not related to later paternal distress [12]. A key limitation of this study is that maternal distress was not adjusted for in the paternal distress model and vice versa. Maternal psychological distress might confound father–child transactional relationships, as family members’ mental health is linked for a number of reasons [26]. Fathers’ distress is correlated with mothers’ distress because individuals with emotional difficulties are likely to choose partners with similar problems [1, 26], or simply because both parents are exposed, as partners, to the same stressors. A related limitation was that, other than child gender, there was no adjustment for either child or family characteristics.

The third study, by Villarreal and Nelson [13], investigated the bidirectional relationships between paternal distress, maternal distress and child internalising difficulties, at around ages 6, 8 and 10, using the same NICHD dataset. Transactional associations were identified between mothers’ and children’s internalising problems (adjusting for paternal internalising symptoms, household income and child’s gender) across the 5 years. But, with regard to fathers, there was only one significant child internalising effect, from age 8 to age 10. There were no father effects, which conflicts with the findings of Gross et al. [11] and Fanti et al. [12] who found father effects on internalising problems.

Taking all this together, the research literature exploring the bi-directional relationships between paternal psychological distress and child difficulties has inconsistent findings. It also has some important limitations. For example, key potential confounding variables, such as maternal distress and prior child mental health problems, have not been adequately controlled for in all studies. Furthermore, most of the research has focused only on childhood, meaning that the evidence regarding the reciprocal paths in adolescence is limited.

The aim of the present study, therefore, was to evaluate the reciprocal associations between father’s psychological distress and child’s emotional and behavioural problems, across childhood and adolescence (ages 3–14), using large-scale longitudinal data from the UK’s Millennium Cohort Study. Psychological distress refers to the overall emotional state of a person at a given time [27]. It may result from symptoms of both anxiety and depression. Though it does not constitute a specific mental health problem, high levels of psychological distress may indicate an underlying psychiatric condition. Those individuals experiencing increased psychological distress may face difficulties in coping effectively with their daily lives [27].

Both biological fathers and stepfathers were included in the sample, unlike prior studies, which included only biological fathers and, therefore, could not extend findings to stepfathers, adoptive or foster fathers [11–13, 16]. We analysed these data using cross-lagged path models that allow for testing these associations over time whilst controlling for prior and concurrent symptoms in children and fathers. We controlled for important confounders, such as child gender, since girls are more likely to experience emotional difficulties and boys are more likely to experience behavioural difficulties [28]. Moreover, we accounted for maternal psychological distress, as it is not only an important risk factor for the development of child difficulties, but it is also linked to paternal psychological distress due to assortative mating [1–4, 26, 29]. Socio-demographic factors, such as household income, ethnicity and educational level, shown to be related to the psychological well-being of both adults and children, were also included in the models [30–35]. Lastly, we controlled for paternal biological status, meaning whether the father living in the same household as the child was the biological father or not. Children who undergo family structure changes are at increased risk for developing emotional and behavioural difficulties. At the same time, stepfathers have been shown to experience elevated levels of psychological distress [36, 37].

## Methods

### Participants

To address the research aim, data from the UK’s Millennium Cohort Study (MCS) were used (https://www.cls.ioe.ac.uk/mcs). The MCS is an on-going birth cohort survey, which includes information on 19,243 UK families (19,517 children) who had a child born in 2000–2002. Participating families have been disproportionately selected, to ensure that UK minority groups and disadvantaged wards are sufficiently represented [38]. Sweeps 1–6 took place when the children were aged 9 months, and 3, 5, 7, 11 and 14 years, respectively. A total of 18,552 families were involved in sweep 1, 15,590 in sweep 2, 15,246 in sweep 3, 13,857 in sweep 4, 13,287 in sweep 5 and, finally, 11,726 in sweep 6. Ethical approval for the MCS has been obtained from NHS Multi-Centre Ethics Committees. Furthermore, parents gave informed consent and children (at the ages of 11 and 14) informed assent. The present investigation has been approved by the IOE Research Ethics Committee.

The analytic sample of this study comprises children and their fathers who met the following criteria: (1) were either a singleton or a first-born twin or triplet, (2) had a father or father figure resident in at least one sweep (2–6)^1^ (children who lived across all sweeps in single-mother families were excluded), (3) had data on father’s or father figure’s psychological distress from at least one sweep (2–6) and (4) had an emotional and behavioural difficulties score in at least one of the available sweeps (2–6). This resulted in an analytic sample of 13,105 children (49.2% of whom were female; biological fathers ranged from 97.6% in sweep 2 to 89.4% in sweep 6).

### Measures

*Paternal psychological distress* was measured with the 6-item Kessler Psychological Distress scale (K-6) in sweeps 2–6, an inventory for which good psychometric properties have been reported [39]. K-6 is a self-administered measure of emotional state and overall levels of distress. It evaluates depressive and anxiety symptoms with questions such as “During the past 30 days, about how often did you feel hopeless?” It includes six items in total, which are rated on a five-point scale, ranging from “none of the time” to “all the time”. Responses are added to create a final score, ranging from 0 to 24, with higher values indicating more difficulties. In the analytic sample, Cronbach’s alphas were .81, .82, .83, .86 and .85 across sweeps 2–6, respectively.

*Child behavioural/emotional difficulties* were measured with the maternal-reported Strengths and Difficulties Questionnaire (SDQ) in sweeps 2–6 [40]. The SDQ includes 25 items, which are answered on a scale 0–2, with 0 corresponding to “not true”, 1 to “somewhat true” and 2 to “certainly true”. There are five sub-scales, consisting of five items each: emotional symptoms, conduct problems, hyperactivity/inattention, peer relations and prosocial behaviour (the ‘strengths’ scale). The four difficulties subscales were included in the present analysis. Each of the sub-scale scores range from 0 to 10, with higher scores indicating more problem behaviours. Across sweeps 2–6, respectively, Cronbach’s alphas, for the emotional domain, were .57, .59, .64, .70 and .72; for the conduct domain, they were .69, .64, .59, .61 and .65; for the hyperactivity domain, they were .73, .77, .79, .79 and .77; lastly, for the peer domain, they were .53, .51, .57, .64 and .62.

*Key covariates* were selected according to the research literature on relationships between paternal psychological distress and child emotional and behavioural difficulties. These included child gender, child ethnicity, paternal educational level, paternal biological status, poverty and maternal psychological distress [1–4, 26, 28–37]. Maternal psychological distress was also measured with the K-6 scale; Cronbach’s alphas across sweeps 2–5 were .86, .87, .87 and .89, respectively. Poverty was also measured at sweeps 3–6, with a binary variable showing whether the family income was below, or above, 60% of the UK’s household median income. Fathers’ biological status was assessed at sweeps 4 and 6, with a binary variable which stated whether the father living in the same household as the child was his/her biological father or not.^2^ Finally, binary variables were used to control for child ethnicity (whether the child was White or not) and paternal educational level (whether the father had graduated from university or not). As paternal educational level was found to vary between sweeps only for a very small number of fathers, information available from the latest sweep was used.

### Statistical analysis

**Fig. 1:**
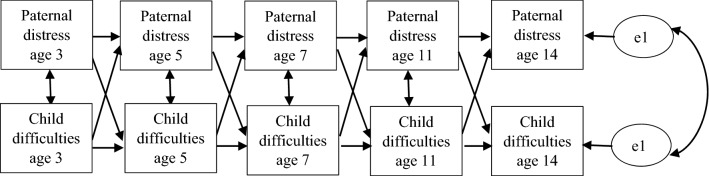
Cross-lagged model for paternal distress and child problem behaviour. Control variables included maternal distress (sweeps 2–5), poverty (sweeps 3–6), paternal education (latest available sweep), paternal biological status (sweeps 4 and 6), child ethnicity and child gender. The control variables predicted both paternal distress and child difficulties in every sweep

First, all study variables were compared between the analytic and non-analytic samples to describe the sample and assess potential sample bias. Thereafter, for the analytic sample, correlations between offspring, paternal and maternal mental health were explored. Finally, cross-lagged structural equation path models (SEM) across all sweeps were run separately for each SDQ domain (emotional symptoms, conduct problems, hyperactivity/inattention and peer relations). Models were at first run without including any covariates; these were added in the second step (Fig. 1). In the case of a cross-lagged effect being found, the formula recommended by Clogg et al. [41] *z* = *β*_1_ − *β*_2_/√ (SE*β*_1_)^2^ + √ (SE*β*_2_)^2^ was used to test which effect (child or father) was stronger.

In the MCS, children and their families living in disadvantaged wards were over-sampled, as were children and their families in areas in England with high proportions of children from ethnic minority backgrounds. There were nine strata in total for the whole of the UK: England ethnic, England advantaged, England disadvantaged, Scotland advantaged, Scotland disadvantaged, Wales advantaged, Wales disadvantaged, Northern Ireland advantaged and Northern Ireland disadvantaged. Furthermore, there were the issues of systematic attrition and non-response [42]. To control for these factors that could generate bias, data were weighted with the use of clustering, stratification and weight variables.

Finally, missing data were treated using full information maximum likelihood (FIML), which allowed us to include our full sample (13,105 cases) in each model. Regarding paternal distress, the percentages of cases with missing data in the analytic sample were 24.7%, 28.4%, 36.6%, 37.4% and 49.5% across sweeps 2–6, respectively. For the child’s total emotional/behavioural problems score, the percentages of cases with missing data in the analytic sample were 10.1%, 14.3%, 20.1%, 23.5% and 31% across sweeps 2–6, respectively. All models were run in STATA 16.

## Results

### Descriptive statistics

Comparison of the descriptive information between the analytic and the non-analytic samples demonstrated some sample selection bias, with those in the analytic sample being from more advantaged backgrounds (Table 1). For all the SDQ domains and across all sweeps, the children in the analytic sample exhibited significantly fewer difficulties. Similarly, maternal distress was consistently lower. Paternal distress was lower in the analytic sample, but we were unable to test whether this was statistically significant given that the vast majority of MCS fathers with valid scores were included in the analysis, and only a few were in the non-analytic sample.^3^

**Table 1:** Descriptives of study variables in the analytic sample and in the non-analytic sample (unweighted data)

Categorical variables	Analytic sample (*n* = 13,105)		Non-analytic sample (*n* = 6138)		
	*N*	%	*n*	%	*χ*^2a^
Girl	6446	49.2	2901	47.3	6.197*
White ethnicity (child)	9801	81.7	4123	82.0	ns
Father is university-educated	4909	38.4	233	18.4	198.863***
Age 3					
Poverty	3183	25.6	1903	64.6	1638.043***
Age 5 years					
Poverty	2969	25.5	1623	67.1	1574.843***
Age 7 years					
Poverty	2470	23	1132	60.4	1096.915***
Biological father	9058	94.2	382	86.8	40.002***
Age 11 years					
Poverty	1894	18.3	900	51.7	939.700***
Age 14 years					
Poverty	2118	22.7	893	61.3	930.927***
Biological father	6448	89.4	216	85	4.881*

Continuous variables	*N*	Mean (SD)	*N*	Mean (SD)	*t*^b^
Age 3 years					
Child emotional symptoms	12,029	1.33 (1.48)	2707	1.62 (1.68)	8.221***
Child conduct problems	12,050	2.72 (2.02)	2713	3.33 (2.24)	12.985***
Child hyperactivity	11,949	3.82 (2.33)	2676	4.40 (2.47)	10.981***
Child peer problems	11,958	1.50 (1.58)	2681	1.83 (1.66)	9.316***
Paternal distress	9865	2.87 (3.13)	43	3.91 (4.55)	–
Maternal distress	11,140	3.10 (3.58)	2343	4.15 (4.44)	10.764***
Age 5 years					
Child emotional symptoms	11,317	1.33 (1.55)	2053	1.64 (1.76)	7.534***
Child conduct problems	11,328	1.42 (1.45)	2060	1.94 (1.72)	12.899***
Child hyperactivity	11,272	3.18 (2.33)	2037	3.88 (2.50)	11.721***
Child peer problems	11,299	1.09 (1.41)	2059	1.53 (1.62)	11.500***
Paternal distress	9384	2.95 (3.34)	13	3.31 (2.84)	–
Maternal distress	10,950	2.96 (3.63)	1927	4.08 (4.57)	10.215***
Age 7 years					
Child emotional symptoms	10,527	1.47 (1.71)	1784	1.86 (1.98)	7.759***
Child conduct problems	10,545	1.30 (1.48)	1793	1.81 (1.74)	11.599***
Child hyperactivity	10,520	3.25 (2.49)	1779	3.95 (2.63)	10.449***
Child peer problems	10,530	1.15 (1.50)	1789	1.62 (1.76)	10.721***
Paternal distress	8310	2.95 (3.41)	4	3.25 (2.75)	–
Maternal distress	10,210	2.93 (3.63)	1681	4.09 (4.72)	9.636***
Age 11 years					
Child emotional symptoms	10,055	1.80 (1.94)	1639	2.22 (2.24)	7.161***
Child conduct problems	10,053	1.29 (1.50)	1641	1.82 (1.79)	11.273***
Child hyperactivity	10,037	3.00 (2.42)	1634	3.70 (2.57)	10.281***
Child peer problems	10,061	1.29 (1.64)	1640	1.76 (1.87)	9.565***
Paternal distress	8203	3.85 (3.90)	4	5.00 (4.24)	–
Maternal distress	9904	3.79 (4.19)	1931	5.44 (5.31)	12.841***
Age 14 years					
Child emotional symptoms	9051	1.96 (2.09)	1389	2.48 (2.31)	7.981***
Child conduct problems	9054	1.32 (1.55)	1389	1.80 (1.82)	9.356***
Child hyperactivity	9050	2.88 (2.36)	1387	3.52 (2.51)	8.959***
Child peer problems	9055	1.65 (1.78)	1391	2.21 (1.94)	10.226***
Paternal distress	6612	363 (3.60)	2	3.50 (3.54)	–
Maternal distress	8526	4.15 (4.07)	1228	5.35 (4.86)	8.189***

*p < 0.05, **p < 0.01, ***p < 0.001

^a^ Pearson’s Chi-Square

^b^ Independent samples t-test

In terms of socio-demographic characteristics, the percentage of university-educated and biological fathers in the analytic sample was significantly higher. Moreover, families in the analytic sample were less likely to be below the poverty line, across all sweeps, and children were more likely to be female. No differences were found regarding ethnicity.

### Correlations

Bivariate correlations were run to investigate the associations between paternal psychological distress, maternal psychological distress and child difficulties. As expected, all correlations were significant and their effect sizes varied from weak to moderate (Appendix, Tables 3, 4, 5, 6, and 7). Correlations between paternal psychological distress and child behavioural and emotional difficulties tended to be quite weak, ranging from 0.07 to 0.19. Correlations between paternal and maternal psychological distress were also weak, ranging from 0.11 to 0.22. Finally, correlations between maternal distress and child difficulties were weak or moderate, ranging from 0.15 to 0.39.

### SEM results

First, models for each SDQ scale were run without accounting for confounding variables (not shown). These models fitted the data satisfactorily, with the Comparative Fit Index (CFI) ranging from 0.90 to 0.93, the Tucker-Lewis Index (TLI) ranging from 0.81 to 0.86 and the Root Mean Squared Error of Approximation (RMSEA) ranging from 0.088 to 0.098.^4^ After adjusting for covariates, model fit improved, with the CFI ranging from 0.91 to 0.93, the TLI from 0.84 to 0.88 and the RMSEA from 0.045 to 0.051 (Table 2). For emotional symptoms (CFI = 0.92, TLI = 0.86, RMSEA = 0.045), there were significant father effects across all sweeps, with elevated paternal distress being associated with more child emotional symptoms at subsequent sweeps, adjusting for the child’s prior emotional difficulties. There were no significant child effects. Similarly, regarding the peer domain (CFI = 0.91, TLI = 0.84, RMSEA = 0.047), higher levels of paternal distress were consistently related to more child peer difficulties at subsequent sweeps. In adolescence, higher peer relations problems at age 11 predicted increased paternal distress at age 14. To test whether the paternal or child effect in adolescence was stronger, we tested which of the two coefficients was larger using the formula, *z* = *β*_1_ − *β*_2_/√ (SE*β*_1_)^2^ + √ (SE*β*_2_)^2^. It was found that *z* = 1.43, meaning that father and child effects did not differ significantly in size. Moreover, there were father effects at age 5 and 11 on conduct problems at ages 7 and 14, respectively (CFI = 0.91, TLI = 0.84, RMSEA = 0.051). Child conduct problems at the age of 3 were related to more paternal distress at the age of 5. Father effects were also found on child hyperactivity (CFI = 0.93, TLI = 0.87, RMSEA = 0.050) between 3 and 5 years and between 11 and 14 years. In addition, offspring difficulties at the age of 7 were related to increased paternal distress at the age of 11. For the cross-sectional relationships, regarding the emotional and hyperactivity domains, paternal distress and offspring difficulties were significantly related only at the ages of 3, 11 and 14. When it comes to the remaining two domains, conduct and peer, they were significantly associated at the ages of 3, 7, 11 and 14. There were no significant links between them at the age of 5.

**Table 2:** Cross-lagged model results (unstandardized coefficients, standard errors and standardized coefficients) adjusted for covariates

Regression paths	Emotional symptoms			Conduct problems			Hyperactivity/inattention			Peer relations		
	*B*	SE	*β*	*B*	SE	*β*	*B*	SE	*β*	*B*	SE	*β*
Stability in paternal psychological distress over time												
Age 3 → age 5	0.59***	0.01	0.55	0.59***	0.01	0.55	0.59***	0.01	0.55	0.59***	0.01	0.55
Age 5 → age 7	0.59***	0.01	0.58	0.59***	0.01	0.58	0.59***	0.01	0.58	0.59***	0.01	0.58
Age 7 → age 11	0.64***	0.01	0.56	0.64***	0.01	0.56	0.64***	0.01	0.56	0.64***	0.01	0.56
Age 11 → age 14	0.57***	0.01	0.61	0.57***	0.01	0.61	0.57***	0.01	0.61	0.57***	0.01	0.61
Stability in child difficulties over time												
Age 3 → age 5	0.41***	0.009	0.39	0.32***	0.006	0.44	0.52***	0.008	0.63	0.32***	0.008	0.36
Age 5 → age 7	0.51***	0.009	0.46	0.56***	0.008	0.55	0.68***	0.008	0.63	0.53***	0.009	0.50
Age 7 → age 11	0.50***	0.01	0.45	0.53***	0.009	0.52	0.61***	0.007	0.64	0.51***	0.01	0.47
Age 11 → age 14	0.52***	0.01	0.48	0.59***	0.009	0.57	0.62***	0.008	0.52	0.57***	0.01	0.53
Cross-sectional relationships (covariance) between paternal psychological distress and child difficulties												
Age 3	0.45***	0.05	0.10	0.70***	0.06	0.11	0.60***	0.07	0.08	0.49***	0.05	0.10
Age 5	− 0.01	0.04	− 0.004	0.05	0.04	0.01	0.09	0.06	0.02	0.05	0.04	0.01
Age 7	0.08	0.05	0.02	0.11**	0.04	0.03	0.10	0.06	0.02	0.08*	0.04	0.02
Age 11	0.24***	0.06	0.05	0.18***	0.05	0.05	0.21**	0.07	0.04	0.17**	0.05	0.04
Age 14	0.30***	0.06	0.06	0.20***	0.05	0.06	0.24***	0.06	0.05	0.13*	0.05	0.03
Cross-lagged relationships between paternal psychological distress and child difficulties												
PD_age3_ → CD_age5_	0.02***	0.005	0.04	0.007	0.004	0.01	0.02**	0.006	0.02	0.01**	0.004	0.03
CD_age3_ → PD_age5_	0.008	0.02	0.003	0.05**	0.02	0.03	0.02	0.01	0.01	0.0008	0.02	0.0003
PD_age5_ → CD_age7_	0.01*	0.005	0.02	0.01*	0.004	0.02	0.007	0.006	0.003	0.01**	0.004	0.03
CD_age5_ → PD_age7_	0.03	0.02	0.01	0.006	0.02	0.002	0.02	0.01	0.01	0.03	0.02	0.01
PD_age7_ → CD_age11_	0.02**	0.005	0.03	− 0.0006	0.004	− 0.001	− 0.002	0.006	0.01	0.01*	0.005	0.03
CD_age7_ → PD_age11_	0.008	0.02	0.004	0.02	0.03	0.008	0.04*	0.02	0.02	0.05	0.03	0.02
PD_age11_ → CD_age14_	0.02**	0.005	0.03	0.02***	0.004	0.04	0.01**	0.05	0.02	0.02***	0.005	0.04
CD_age11_ → PD_age14_	0.02	0.02	0.01	0.04	0.03	0.02	− 0.004	0.02	0.003	0.05*	0.02	0.02

*β* standardized beta coefficient, *PD* paternal psychological distress, *CD* child difficulties

*p < 0.05, **p < 0.01, ***p < 0.001

Regarding covariates, fathers and children presented more difficulties if maternal distress was high and they were living below the poverty line. Across all ages, children living with stepfathers or mothers’ partners displayed more difficulties, while biological fathers experienced more psychological distress. Fathers without a university degree appeared to have higher psychological distress when their child was age 14. Furthermore, children were more likely to exhibit problems if they lived with a father without a university degree. Additionally, female compared to male adolescents were more likely to show emotional difficulties at 11 and 14 years. Males, at all ages, were at higher risk for peer and hyperactivity difficulties. In childhood and early adolescence (5, 7 and 11 years), they were also more likely to present with conduct problems. Finally, child ethnicity did not have any significant associations with paternal distress or child difficulties.

### Sensitivity analyses

The criteria used to define the analytic sample involved having a father or father-figure present in the household in at least one sweep, but not in every sweep. Therefore, FIML may have predicted paternal distress scores in study sweeps where there was no father or father-figure living with the family. We conducted a sensitivity analysis to test whether our findings may differ if the analytic sample included only households who had a resident father/father-figure in every sweep, in addition to the other inclusion criteria. This resulted in a sample of 5412 cases. Compared to the main analytic sample, a few differences were identified. In particular, for emotional symptoms, the father effects at the ages of 3 and 5 ceased to exist. Similarly, for the peer domain, paternal distress at the age of 7 no longer predicted child difficulties. Importantly, the cross-lagged paths between the ages of 11 and 14 remained while, again, neither of them was stronger than the other as *z* = 1.17. Regarding conduct problems, there was no father effect at the age of 5. However, another set of cross-lagged effects was found to occur between ages 3 and 5. Clogg’s formula showed that none of these paths was stronger than the other (*z* = 0.56). Finally, for the hyperactivity domain, child difficulties at the age of 7 no longer predicted paternal distress (results available in the Supplement, table S1).

Furthermore, although most of the fathers in the sample were biological, not all of them were. As one of the possible reasons for father effects is genetic transmission, another sensitivity analysis was run, which included only families that had a biological father present in the household in every sweep, leading to a sample of 5258 families. Households with stepfathers/non-biological fathers therefore were excluded. Using this sample, for emotional difficulties, there were no father influences at ages 3 and 5, as was the case in the main analysis. However, a new set of cross-lagged effects occurred between father distress and child emotional difficulties, between ages 11 and 14. Using Clogg’s formula, it was shown that neither path was stronger than the other (*z* = − 0.87). Regarding conduct problems, paternal distress at age 5 no longer predicted child outcomes. The cross-lagged relationships between ages 3 and 5 were found again (as in the previous supplementary analysis). However, again, father and child effects did not differ significantly in size (*z *= 1.36). With respect to hyperactivity, there was no child effect at age 7. Lastly, there was no path from peer difficulties at age 7 to father distress at age 11; however, father distress at age 7 predicted age 11 peer difficulties. The reciprocal relationships between ages 11 and 14 remained, with no effect being stronger than the other (*z* = 1.92) (results available in the Supplement, table S2).

Overall, compared to the main analysis, there were no unidirectional child effects in either sensitivity analysis. However, across all three sets of analyses, the reciprocal relationship in adolescence between peer problems and paternal distress remained significant. The two sensitivity analyses revealed another reciprocal relationship, this time in early childhood: between conduct problems and paternal distress. Perhaps the most important conclusion from this supplementary work is that in both sensitivity analyses fewer father influences were identified, compared to the main analysis. We note that families not included in the sensitivity analyses are likely to be those facing more challenges, such as divorce. As a result, paternal distress in those families would be expected to be higher, which could largely drive the effects observed in the main analysis. Indeed, the mean paternal distress scores in both sensitivity analyses were lower than those in the main analytic sample. Particularly, for the analysis that included a father/father-figure present in all sweeps, mean (SD) scores were 2.73 (2.83), 2.81 (3.12), 2.81 (3.21), 3.64 (3.68) and 3.57 (3.53), across sweeps 2–6, respectively. In the analysis that included only biological fathers, mean (SD) scores equalled 2.72 (2.83), 2.81 (3.11), 2.80 (3.21), 3.62 (3.66) and 3.55 (3.50), across sweeps 2–6, respectively (descriptive statistics for the sensitivity analyses samples are available upon request).

## Discussion

There is evidence that suggests that paternal psychological distress is related to children’s behavioural and emotional difficulties [8–10] over and above maternal psychological distress. However, little is known about whether paternal psychological distress and child difficulties influence each other, especially over an extended time period. Therefore, this study aimed to explore the reciprocal relationships between paternal psychological distress and offspring behavioural and emotional difficulties across both childhood and adolescence (ages 3–14 years) in a large and nationally representative longitudinal sample. Furthermore, we controlled for many important confounding variables such as maternal psychological distress, family income and paternal biological status.

We found that paternal psychological distress was associated with not only later emotional and behavioural difficulties after adjustment for confounders, but also prior emotional and behavioural difficulties and later paternal psychological distress. Specifically, father’s distress at child’s age 3 was related to more hyperactivity at age 5. At age 5, it was associated with more conduct problems at age 7. At age 11, paternal distress was also related to age 14 conduct problems and hyperactivity. Child effects were fewer and were found for behavioural problems and peer problems but not emotional symptoms. In particular, conduct problems at age 3 were shown to be related to higher paternal distress at age 5. Hyperactivity at age 7 was associated with higher paternal distress at age 11. And peer problems at age 11 were predictive of paternal distress at age 14.

Moreover, we found reciprocal links between paternal psychological distress and offspring peer difficulties in adolescence between ages 11 and 14. These two effects did not differ significantly in size, meaning that the influence of paternal distress on peer problems and that of peer problems on paternal distress were of similar strength. These transactional relationships are partly in line with previous findings from Fanti et al. [12]. In that study, there were bidirectional associations in the internalising domain during early adolescence (11–12 years), but not in later adolescence (12–15 years), when offspring symptoms did not predict paternal distress. Methodological differences could potentially account for this inconsistency, as our study used a more representative sample and adjusted for key background factors as well as maternal psychological distress. What is more, in the study of Fanti et al. [12], internalising difficulties were broadly defined, while our study assessed emotional and peer problems separately, confirming the reciprocal links only for peer problems. If causal, these bidirectional relationships suggest that elevated levels of paternal distress lead adolescents to have poorer peer relations; at the same time, when adolescents experience more peer problems their fathers become more distressed.

One explanation for the influence of peer problems on father’s distress is that higher levels of peer difficulties might indicate that the child is being bullied (as some items in the peer scale capture peer victimisation), which the father might view as evidence of his shortcomings as a parent, resulting in feelings of inadequacy and low self-esteem. In addition, parents of a child who experiences peer victimisation often feel guilt and worry about their child’s well-being, in turn leading to more distress [44, 45]. What is more, peer victimisation is associated with increased risk of adolescent risky behaviour, which may be a further cause for paternal worry [46, 47]. There are also several reasons why fathers’ psychological distress may predict an increase in adolescents’ peer difficulties. Some research suggests that fathers are particularly good at motivating their children to explore new and unfamiliar situations [48], thus promoting their independence and healthy risk-taking, attributes that help children to manage new social situations and form peer relationships, a significant and important part of the adolescent experience [49]. Fathers who experience high levels of distress tend to be less involved and engaged with their children [50, 51]. Consequently, their offspring might miss out on the aforementioned father–child interactions and their associated benefits for social development. Furthermore, psychological distress and loneliness in adults are linked [52], meaning that fathers with high levels of psychological distress may have few or weak friendships or social relationships. Therefore, their children may lack the broader social networks or indeed the opportunities to build their social skills by observing their parents’ social interactions, both of which could hinder them from developing healthy peer relationships [53].

Overall, in line with past research, a fair amount of father effects was identified. Most of them concerned emotional and peer problems, while there was a lesser number of effects on behavioural problems. The greater association between paternal psychological distress and emotional and peer problems was expected due to genetic transmission links [54] in biological families, the vast majority of our sample. Additionally, it is notable that paternal distress at the age of 11 predicted offspring difficulties at the age of 14 in all domains. Possibly, as children grow up and enter adolescence, they become more perceptive and aware of their family environment and of their parents’ emotions. Consequently, they are more likely to be affected by them [55], especially because in adolescence fathers are also more engaged independently with them. Moreover, in our sample, at the age 11 sweep, fathers experienced the highest levels of distress, significantly higher than in previous sweeps, which could perhaps also account for the significant father effects observed in adolescence.

Fewer child effects were found with most occurring in the behavioural domain. Behavioural problems typically cause more family disruption than emotional problems do, and as a consequence they are more likely to adversely influence fathers [56]. Our findings are mainly in line with those from Fanti et al. (2013). Particularly, for externalising difficulties, both that study and ours found father and child effects for similar developmental stages. Regarding emotional and peer difficulties, the outcomes from both studies show that paternal distress always predicted subsequent child problems. However, our findings for these problems are not in line with Villarreal and Nelson [13], who reported only one child effect from 8 to 10 years and no father effects. A possible explanation for this inconsistency is that Villarreal and Nelson [13] assessed paternal anxiety symptoms, while our study utilized a general measure of psychological distress.

Our study also has some limitations. First, the identification of father and child effects using cross-lagged models is limited by the specific timepoints at which measures of mental health were taken. Some effects may be shorter term (or longer term) than can be measured using the available data. Second, the lagged parameters that are obtained with the cross-lagged panel modelling approach that we adopted do not represent the actual within-person relationships over time, and this may lead to erroneous conclusions regarding the presence, predominance and sign of causal influences [57]. Third, the time-points measured were not equidistant, as the year differences between them are 2, 2, 4 and 3 years, respectively. Furthermore, child assessments were based solely on one reporter, usually the mother, which might limit their accuracy through reporter-bias. Especially when it comes to the relationship between child difficulties and maternal distress, mothers who experienced high levels of distress might have inflated their children’s adjustment problems. Moreover, even though the general sample was large and representative, the participants of our analytic sample tended to come from higher socio-economic backgrounds and overall displayed fewer difficulties, meaning that conclusions might not apply to a higher-risk sample. In addition, Cronbach’s alphas for some of the sweeps in the emotional, conduct and peer domains ranged from 0.5 to 0.6. Lastly, effect sizes for paternal distress and offspring difficulties were small (0.01–0.05).

Future studies could aim to address some of these limitations, and expand our results by evaluating potential moderators, such as child gender or ethnicity. They could also assess the causal pathways that might explain these associations, such as parenting practices or genetic links. Most importantly, it would be useful to explore the reasons why peer problems appear to raise father’s distress in adolescence but not in the early years. The reasons for child behaviour effects on fathers is an area where little research has been conducted. Possible pathways that could be examined include engagement in delinquent or risky behaviours, which start to occur during adolescence [46, 47].

In conclusion, this study highlights the likely adverse influence of paternal psychological distress on offspring behavioural and emotional development as well as the role of child emotional and behavioural problems in paternal distress. It also shows that reciprocal relationships occur between fathers' psychological distress and adolescents’ peer problems. Importantly, all the associations found were independent of the effects of third factors, including maternal distress and family income. Our findings underline the importance of preventing mental health problems in fathers as well as supporting fathers who experience them. Moreover, they highlight the need to support the well-being of the children of fathers who experience psychological distress with prevention or management strategies, as they may be at increased risk for developing emotional and behavioural difficulties.

### Electronic supplementary material

Below is the link to the electronic supplementary material.

Supplementary file1 (RTF 369 kb)

## Data Availability

N/A.

## Appendix

See Tables 3, 4, 5, 6 and 7.

**Table 3:** Bivariate correlations between paternal and maternal distress

	1	2	3	4	5	6	7	8	9	10
1. S2 paternal distress	1									
2. S3 paternal distress	0.55	1								
3. S4 paternal distress	0.51	0.58	1							
4. S5 paternal distress	0.43	0.49	0.55	1						
5. S6 paternal distress	0.42	0.46	0.52	0.61	1					
6. S2 maternal distress	0.18	0.15	0.13	0.13	0.14	1				
7. S3 maternal distress	0.12	0.18	0.16	0.15	0.14	0.56	1			
8. S4 maternal distress	0.12	0.15	0.19	0.16	0.11	0.53	0.59	1		
9. S5 maternal distress	0.14	0.13	0.15	0.22	0.17	0.48	0.51	0.55	1	
10. S6 maternal distress	0.11	0.13	0.13	0.14	0.18	0.46	0.48	0.51	0.61	1

All correlation are significant at *p* < 0.001 level

*S2* sweep 2, *S3* sweep 3, *S4* sweep 4, *S5* sweep 5, *S6* sweep 6

**Table 4:** Bivariate correlations between paternal, maternal distress and emotional symptoms

	S2 paternal distress	S3 paternal distress	S4 paternal distress	S5 paternal distress	S6 paternal distress	S2 maternal distress	S3 maternal distress	S4 maternal distress	S5 maternal distress	S6 maternal distress	S2 emotional	S3 emotional	S4 emotional	S5 emotional	S6 emotional
S2 emotional	0.09	0.08	0.07	0.09	0.08	0.23	0.19	0.17	0.19	0.17	1				
S3 emotional	0.10	0.08	0.09	0.10	0.10	0.21	0.27	0.20	0.22	0.21	0.43	1			
S4 emotional	0.10	0.08	0.11	0.09	0.08	0.22	0.24	0.30	0.24	0.24	0.34	0.50	1		
S5 emotional	0.11	0.09	0.11	0.14	0.12	0.23	0.24	0.25	0.34	0.30	0.26	0.38	0.50	1	
S6 emotional	0.10	0.09	0.11	0.13	0.15	0.20	0.21	0.22	0.27	0.31	0.23	0.33	0.40	0.54	1

All correlation are significant at *p* < 0.001 level

*S2* sweep 2, *S3* sweep 3, *S4* sweep 4, *S5* sweep 5, *S6* sweep 6, *emotional* child emotional symptoms

**Table 5:** Bi-variate correlations between paternal, maternal distress and conduct problems

	S2 paternal distress	S3 paternal distress	S4 paternal distress	S5 paternal distress	S6 paternal distress	S2 maternal distress	S3 maternal distress	S4 maternal distress	S5 maternal distress	S6 maternal distress	S2 conduct	S3 conduct	S4 conduct	S5 conduct	S6 conduct
S2 conduct	0.11	0.12	0.11	0.10	0.08	0.28	0.23	0.21	0.22	0.20	1				
S3 conduct	0.09	0.11	0.11	0.11	0.10	0.21	0.26	0.23	0.21	0.20	0.49	1			
S4 conduct	0.10	0.11	0.12	0.11	0.09	0.20	0.23	0.27	0.23	0.21	0.43	0.59	1		
S5 conduct	0.09	0.08	0.09	0.13	0.12	0.20	0.20	0.23	0.29	0.23	0.39	0.48	0.56	1	
S6 conduct	0.09	0.10	0.14	0.14	0.16	0.17	0.18	0.21	0.25	0.24	0.33	0.41	0.48	0.61	1

All correlation are significant at *p* < 0.001 level

*S2* sweep 2, *S3* sweep 3, *S4* sweep 4, *S5* sweep 5, *S6* sweep 6, *conduct* child conduct problems

**Table 6:** Bi-variate correlations between paternal, maternal distress and child hyperactivity/inattention

	S2 paternal distress	S3 paternal distress	S4 paternal distress	S5 paternal distress	S6 paternal distress	S2 maternal distress	S3 maternal distress	S4 maternal distress	S5 maternal distress	S6 maternal distress	S2 hyperactivity	S3 hyperactivity	S4 hyperactivity	S5 hyperactivity	S6 hyperactivity
S2 hyperactivity	0.08	0.09	0.09	0.08	0.07	0.21	0.17	0.17	0.17	0.18	1				
S3 hyperactivity	0.09	0.10	0.11	0.09	0.08	0.21	0.23	0.21	0.21	0.19	0.57	1			
S4 hyperactivity	0.10	0.09	0.10	0.11	0.09	0.18	0.20	0.22	0.21	0.18	0.51	0.68	1		
S5 hyperactivity	0.08	0.09	0.09	0.13	0.10	0.20	0.20	0.22	0.28	0.22	0.43	0.57	0.67	1	
S6 hyperactivity	0.09	0.07	0.09	0.13	0.13	0.18	0.18	0.20	0.24	0.24	0.40	0.50	0.58	0.68	1

All correlation are significant at *p* < 0.001 level

*S2* sweep 2, *S3* sweep 3, *S4* sweep 4, *S5* sweep 5, *S6* sweep 6, *hyperactivity* child hyperactivity/inattention

**Table 7:** Bi-variate correlations between paternal, maternal distress and child peer relations

	S2 paternal distress	S3 paternal distress	S4 paternal distress	S5 paternal distress	S6 paternal distress	S2 maternal distress	S3 maternal distress	S4 maternal distress	S5 maternal distress	S6 maternal distress	S2 peer	S3 peer	S4 peer	S5 peer	S6 peer
S2 peer	0.09	0.07	0.07	0.07	0.08	0.19	0.16	0.16	0.17	0.15	1				
S3 peer	0.08	0.09	0.09	0.08	0.10	0.19	0.22	0.18	0.20	0.18	0.40	1			
S4 peer	0.09	0.09	0.12	0.11	0.11	0.20	0.22	0.25	0.24	0.19	0.34	0.53	1		
S5 peer	0.09	0.09	0.09	0.14	0.12	0.20	0.19	0.20	0.26	0.22	0.27	0.39	0.50	1	
S6 peer	0.08	0.10	0.11	0.13	0.14	0.18	0.18	0.18	0.34	0.23	0.25	0.35	0.43	0.57	1

All correlation are significant at *p* < 0.001 level

*S2* sweep 2, *S3* sweep 3, *S4* sweep 4, *S5* sweep 5, *S6* sweep 6, *peer* child peer relation problems

## Footnotes

1 Out of the total MCS sample at sweeps 2–6, 14,504 children (75.4%) had a father/father figure present in the household in at least one sweep. After applying the exclusion criteria, 13,105 of fathers (68.1%) were included in the analytic sample.

2 Examination of the covariates revealed that there was high collinearity between the paternal biological status variables across the sweeps, based on the high variance inflation factor (VIF > 4). For this reason, paternal biological status in sweeps 3 and 5 were removed from the analysis. Therefore, biological status was controlled for only in sweeps 4 and 6.

3 A repeated-measures ANOVA was conducted to compare the levels of paternal psychological distress between sweeps 2–6 in the analytic sample. Results revealed that there was a significant difference between sweeps, *F*(3.73, 14,994.70) = 160.943, *p* < *0.001*. Pairwise comparisons showed that paternal distress at sweeps 5 and 6 was significantly higher than paternal distress at sweeps 2, 3 and 4.

4 It is recommended that good model fit is achieved when CFI ≥ .95, TLI ≥ .95 and RMSEA < 0.07 [43].
