# Supplementary material for: Reciprocal relationships between paternal psychological distress and child internalising and externalising difficulties from 3 to 14 years: a cross-lagged analysis
Source: Eur Child Adolesc Psychiatry. 2020 Sep 17;30(11):1695–708. doi: 10.1007/s00787-020-01642-0 (PMC8558163; doi:10.1007/s00787-020-01642-0)
Supplement: Supplementary file 1 — Supplementary file1 (RTF 369 kb) [file 787_2020_1642_MOESM1_ESM.rtf]

Table S1 Cross-lagged model results for sample with resident father/father figure in every sweep (unstandardized coefficients, standard errors and standardized coefficients), adjusted for covariates
Regression paths	Emotional symptoms	Conduct problems	Hyperactivity 	Peer relations	
	b	SE	â 	b	SE	â	b	SE	â	b	SE	â	
Stability in paternal psychological distress over time										
Age 3 → Age 5
Age 5 → Age 7
Age 7 → Age 11
Age 11 → Age 14	0.60***
0.59***
0.63***
0.58***	0.01
0.01
0.01
0.01	0.54
0.57
0.55
0.61	0.60***
0.59***
0.63***
0.58***	0.01
0.01
0.01
0.01	0.54
0.57
0.55
0.61	0.60***
0.59***
0.63***
0.58***	0.01
0.01
0.01
0.01	0.54
0.57
0.55
0.61	0.60***
0.59***
0.63***
0.58***	0.01
0.01
0.01
0.01	0.54
0.57
0.55
0.61	
Stability in child difficulties over time										
Age 3 → Age 5
Age 5 → Age 7
Age 7 → Age 11
Age 11 → Age 14	0.42***
0.52***
0.50***
0.53***	0.01
0.01
0.01
0.01	0.39
0.47
0.45
0.50	0.30***
0.53***
0.50***
0.56***	0.009
0.01
0.01
0.01	0.42
0.54
0.50
0.54	0.54***
0.68***
0.60***
0.62***	0.01
0.01
0.01
0.01	0.54
0.64
0.62
0.63	0.33***
0.53***
0.49***
0.59***	0.01
0.01
0.01
0.01	0.37
0.50
0.45
0.54	
Cross-sectional relationships (covariance) between paternal psychological distress and child difficulties							
Age 3
Age 5
Age 7
Age 11
Age 14	0.42***
0.03
0.07
0.22**
0.23***	0.06
0.05
0.05
0.07
0.06	0.11
0.009
0.02
0.05
0.05	0.51***
0.03
0.17**
0.21***
0.20***	0.08
0.04
0.04
0.05
0.05	0.09
0.009
0.06
0.06
0.06	0.41***
0.11
0.21**
0.23**
0.22**	0.09
0.07
0.06
0.07
0.06	0.06
0.02
0.05
0.05
0.05	0.31***
0.02
0.03
0.12*
0.11*	0.06
0.05
0.04
0.06
0.05	0.07
0.007
0.008
0.03
0.03	
Cross-lagged relationships between paternal psychological distress and child difficulties							
PDage3 → CDage5	0.01	0.007	0.02	0.02**	0.006	0.04	0.03**	0.009	0.04	0.02**	0.006	0.05	
CDage3 → PDage5	-0.01	0.03	-0.004	0.04*	0.02	0.03	0.01	0.02	0.07	0.02	0.03	0.01	
PDage5 → CDage7	0.006	0.006	0.01	0.003	0.005	0.006	0.0004	0.008	0.0005	0.02**	0.005	0.04	
CDage5 → PDage7	0.01	0.03	0.006	0.02	0.03	0.008	0.003	0.02	0.002	0.008	0.03	0.003	
PDage7 → CDage11	0.02**	0.007	0.04	-0.002	0.005	-0.004	0.0002	0.008	0.0003	0.01	0.006	0.02	
CDage7 → PDage11	0.02	0.03	0.008	-0.001	0.03	-0.0004	0.02	0.02	0.01	0.05	0.03	0.02	
PDage11 → CDage14	0.03***	0.006	0.05	0.02***	0.004	0.05	0.02**	0.06	0.03	0.02***	0.005	0.05	
CDage11 → PDage14	0.04	0.02	0.02	0.01	0.03	0.005	0.004	0.02	0.003	0.08**	0.03	0.03	
*p < .05, **p < .01, ***p < .001. â = standardized beta coefficient. PD=paternal psychological distress, CD = child difficulties                     


Table S2 Cross-lagged model results for sample with biological father in every sweep (unstandardized coefficients, standard errors and standardized coefficients), adjusted for covariates
Regression paths	Emotional symptoms	Conduct problems	Hyperactivity 	Peer relations	
	b	SE	â 	b	SE	â	b	SE	â	b	SE	â	
Stability in paternal psychological distress over time										
Age 3 → Age 5
Age 5 → Age 7
Age 7 → Age 11
Age 11 → Age 14	0.61***
0.59***
0.63***
0.58***	0.01
0.01
0.01
0.01	0.55
0.57
0.55
0.61	0.60***
0.59***
0.63***
0.58***	0.01
0.01
0.01
0.01	0.55
0.57
0.55
0.61	0.61***
0.59***
0.63***
0.58***	0.01
0.01
0.01
0.01	0.55
0.57
0.55
0.61	0.61***
0.59***
0.63***
0.58***	0.01
0.01
0.01
0.01	0.55
0.57
0.55
0.61	
Stability in child difficulties over time										
Age 3 → Age 5
Age 5 → Age 7
Age 7 → Age 11
Age 11 → Age 14	0.42***
0.52***
0.50***
0.53***	0.01
0.01
0.01
0.01	0.39
0.47
0.45
0.49	0.30***
0.53***
0.49***
0.54***	0.009
0.01
0.01
0.01	0.42
0.54
0.49
0.52	0.54***
0.69***
0.60***
0.62***	0.01
0.01
0.01
0.01	0.54
0.64
0.62
0.63	0.33***
0.53***
0.49***
0.58***	0.01
0.01
0.01
0.01	0.37
0.50
0.45
0.53	
Cross-sectional relationships (covariance) between paternal psychological distress and child difficulties							
Age 3
Age 5
Age 7
Age 11
Age 14	0.43***
0.03
0.07
0.22**
0.19**	0.06
0.05
0.05
0.07
0.06	0.11
0.01
0.02
0.05
0.04	0.54***
0.03
0.18***
0.22***
0.20***	0.08
0.04
0.04
0.05
0.04	0.09
0.009
0.06
0.06
0.06	0.43***
0.08
0.22**
0.24**
0.21**	0.10
0.07
0.06
0.07
0.06	0.07
0.02
0.05
0.05
0.05	0.31***
0.03
0.02
0.14*
0.10	0.07
0.05
0.04
0.06
0.05	0.07
0.01
0.008
0.04
0.03	
Cross-lagged relationships between paternal psychological distress and child difficulties							
PDage3 → CDage5	0.01	0.007	0.02	0.02***	0.006	0.05	0.03**	0.01	0.04	0.02***	0.006	0.05	
CDage3 → PDage5	-0.01	0.03	-0.004	0.05*	0.02	0.03	0.02	0.02	0.01	0.04	0.03	0.02	
PDage5 → CDage7	0.006	0.006	0.01	0.002	0.005	0.004	-0.001	0.008	-0.002	0.02**	0.005	0.04	
CDage5 → PDage7	0.01	0.03	0.006	0.02	0.03	0.008	0.01	0.02	0.007	0.01	0.03	0.005	
PDage7 → CDage11	0.02**	0.007	0.04	-0.0007	0.005	-0.002	0.00003	0.008	.00004	0.01	0.006	0.02	
CDage7 → PDage11	0.02	0.03	0.009	-0.006	0.03	-0.002	0.02	0.02	0.01	0.07*	0.03	0.03	
PDage11 → CDage14	0.03**	0.006	0.04	0.02***	0.005	0.05	0.02**	0.06	0.03	0.02***	0.005	0.05	
CDage11 → PDage14	0.04*	0.02	0.02	0.007	0.03	0.003	-0.0006	0.02	-.0004	0.07**	0.03	0.03	
*p < .05, **p < .01, ***p < .001. â = standardized beta coefficient. PD=paternal psychological distress, CD = child difficulties              
